# The World of Organoids: Gastrointestinal Disease Modelling in the Age of 3R and One Health with Specific Relevance to Dogs and Cats

**DOI:** 10.3390/ani12182461

**Published:** 2022-09-18

**Authors:** Georg Csukovich, Barbara Pratscher, Iwan Anton Burgener

**Affiliations:** Small Animal Internal Medicine, Vetmeduni, 1210 Vienna, Austria

**Keywords:** one health, 3R, organoids

## Abstract

**Simple Summary:**

One Health is a concept that describes the interplay between humans, animals, and the environment. This interaction is becoming increasingly important as researchers try to address it in a laboratory setting. This has led to the development of new and highly sophisticated research methods paving the way for animal-free research methods. Within this context, the development of mini-organs, so-called ‘organoids’, is of great significance. These organoids represent entire organs on a laboratory scale and can be established from stem cells. Subsequently, organoids are used to model certain disease states and the interaction of the host with specific harmful organisms. With this review, we give an overview of what disease modelling approaches have already been carried out in the past and where the field might be heading in the future. In the context of One Health, we consider animal models whenever possible, putting a focus on gastrointestinal diseases.

**Abstract:**

One Health describes the importance of considering humans, animals, and the environment in health research. One Health and the 3R concept, i.e., the replacement, reduction, and refinement of animal experimentation, shape today’s research more and more. The development of organoids from many different organs and animals led to the development of highly sophisticated model systems trying to replace animal experiments. Organoids may be used for disease modelling in various ways elucidating the manifold host–pathogen interactions. This review provides an overview of disease modelling approaches using organoids of different kinds with a special focus on animal organoids and gastrointestinal diseases. We also provide an outlook on how the research field of organoids might develop in the coming years and what opportunities organoids hold for in-depth disease modelling and therapeutic interventions.

## 1. Introduction

The concept of ‘One Health’ has become increasingly important over the last few years. In contrast to specific scientific disciplines such as human medicine, veterinary medicine, or environmental sciences, One Health is an approach taking more than one of these factors into account [1]. This also includes the political implications of the surveillance of diseases and the prevention thereof and not only scientific research on pathogens and their interaction with host organisms. COVID-19, for instance, is a very prominent and current example. SARS-CoV-2 infections are diagnosed in humans as well as many different species of animals [2], and viral particles can be found in wastewater [3]. Referring to the global problem of SARS-CoV-2 infections for humans, animals, and environmental contamination, one can appreciate the importance of One Health in a global context. In vitro research methods can neither fully model these complex interactions nor entirely replace animal experimentation but are of great importance to reduce the need for animals in today’s research.

More than 60 years ago, researchers were looking for ways to reduce pain and distress for laboratory animals. In 1959, Russel and Burch first explained the principle of the three Rs (3R), i.e., Replacement, Reduction, and Refinement of animal experimentation [4]. Since then, the 3R principle has been implicitly included in animal welfare laws in the United States of America [5] as well as in Europe [6], and researchers are obliged to consider these laws when planning and carrying out experiments involving live animals. Recently, the Max Planck Society for the Advancement of Science e.V. has taken the next step and expanded the classic 3R principle to the 4R principle, also taking ‘Responsibility’ into account. Researchers commit to using their knowledge in order to further promote animal welfare by engaging in public discourse, improving the social structure of housed experimental animals and expanding the knowledge about the experience of pain, intelligence and consciousness in animals [7]. Animal experimentation is not limited to laboratory mice and rats but also includes other vertebrates such as fish, rabbits, cats, dogs, pigs, and others.

**Figure 1:**
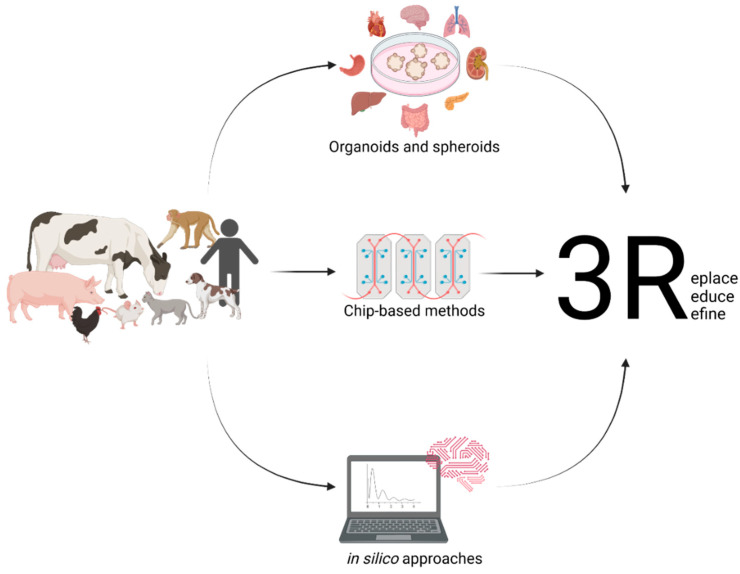
Setting up useful in vitro models from different animals and their various organs and the use of in silico modelling will help to replace the need for animal experimentation.

Dogs, for instance, are mainly used for toxicology studies. In the European Union, the number of dogs used for any scientific purpose for the first time accounted for 17.711 in 2018, adding up to 25.717, including dogs already in use [8]. By far, the number is exceeded by the United States, with them having used 58.511 dogs for research in 2019 [9]. These numbers clearly demonstrate the need for replacing animal experimentation with meaningful in vitro or in silico methods according to the 3Rs (and 4R concept) principle or at least reducing them to an absolute minimum. This leads to the improvement of the state-of-the-art in vitro methods to reduce the animal numbers used for research and minimise the pain experienced during experiments. These comprise but are not limited to the use of classical cell culture models as well as more advanced methods such as three-dimensional model systems such as tumour spheroids, organoids, organ-on-a-chip technologies, or computer-based models such as prediction methods based on artificial intelligence (AI), as previously applied to diabetes [10], cardiovascular disease [11], tuberculosis [12], and drug discovery [13]. Spheroids pose a model of compact three-dimensional cell aggregates consisting of cells at different states, e.g., proliferating, hypoxic, and quiescent, which are generated on non-adherent surfaces. These do not necessarily represent complex organ architecture on a miniature scale [14]. On the other hand, organoids are three-dimensional models of organ systems reflecting organ microanatomy. Due to their stem-cell-originating nature, organoids are usually indefinitely expandable [15,16]. Modelling different organ systems of various animals will help to replace animal experimentation in accordance with the 3Rs (and 4R concept) principle. This leads to an improved understanding of the biological principles in a broader context, as humans and different species of animals may react differently to various irritants (Figure 1). In-depth knowledge of diverse species and their organs is pivotal for research in a One Health context, taking humans, animals, and the environment into account. Thus, this review deals with the importance of organoids for today’s research and provides an overview of different methods for disease modelling and highlights the limitations of organoids, differences between humans and animals and the possible future applications of organoid-based in vitro research.

## 2. The Importance of Organoids for One Health

The establishment of meaningful in vitro systems to model complex diseases is very important. At the moment, the world is progressing from using classical cell culture models to more sophisticated three-dimensional models to investigate the effects of commensal or pathogenic organisms on certain cells/organs of humans, companion animals as well as farm animals. In humans, many different organs are available as organoid systems, e.g., the brain [17], retina [18], salivary gland [19], thyroid [20], lung [21], blood vessels [22] and the heart [23], mammary gland [24], stomach [25], liver [26], kidney [27], pancreas [28], intestine [29,30], fallopian tube [31], endometrium [32], bladder [33] and the prostate [34]. Many of these can be adapted to cancer organoid cultures, and some have been translated to animal organoid models. There are also very sophisticated air–liquid interface models of patient-derived cancer organoids. One of these models even includes the complex tumour microenvironment with immune cells, making it a very attractive and complex model [35]. A lot of work has been undertaken on organoids from companion animals, including the canine and feline intestine [36,37,38,39], the canine and feline liver [40,41], and canine kidney [42], bladder cancer [43], prostate cancer [44], skin [45], and thyroid tissue [46]. These companion animal models are further complemented by organoids derived from farm animals. Among them are primarily intestinal organoids from several species such as pigs, cattle, sheep, horses, and chickens [47], which have recently been reviewed more in-depth elsewhere [48]. In this context, organoids may develop towards a central model connecting the three cornerstones of the One Health concept regarding the physiological and pathophysiological interrelation of human, animal, and environmental health.

Gastrointestinal (GI) diseases do not only affect humans but also constitute a major threat to farm and companion animals and are associated with high costs to healthcare systems and animal owners. Just as in humans, conceivably lethal GI diseases also affect animals. Enteropathogenic viruses and bacteria are frequently responsible for the initiation or further impairment of GI afflictions [49,50,51]. There are numerous examples of the pathogenic organisms involved in the development of health problems in humans as well as animals. Several reviews have recently highlighted the importance of One Health approaches putting surveillance, monitoring, and treatment options in a broader context compared to studies investigating only one aspect of potentially zoonotic pathogens. Due to the fact that some pathogens can survive in the environment or animal products consumed by humans, the transmission routes should be examined more closely.

Especially, enteric pathogens are a major threat in a zoonotic One Health context, including parasites such as helminths [52], *Giardia duodenalis*, *Blastocystis*, and *Cryptosporidium* spp. [53], as well as bacteria such as *Clostridioides difficile* (*C. difficile*) [54,55,56], *Bacillus cereus* sensu lato [57], and *Salmonella* [58], which all affect humans as well as animals. Particularly, the widespread *C. difficile* has been well studied, with the faeces of animals contaminating soil and water with *C. difficile* spores, leading to the spread of the disease to other animals. Alike, the spores from infected humans show up in wastewater, highlighting the importance of *C. difficile* for the environment as well as human and veterinary medicine [59]. This is complemented by reports that animals may be important asymptomatic carriers of toxigenic *C. difficile* [60,61]. Additionally, the co-clustering of isolates from cattle and dogs with isolates from human newborns has been documented, indicating the opportunity for inter-species transmission, either directly or indirectly, via contaminated environments [62]. How food intake shapes gut health has also been reviewed many times. Especially, fermented foods have received a lot of attention because of their ability to substantially change gut microbiota composition and therefore influence physiologic as well as pathologic processes [63].

**Figure 2:**
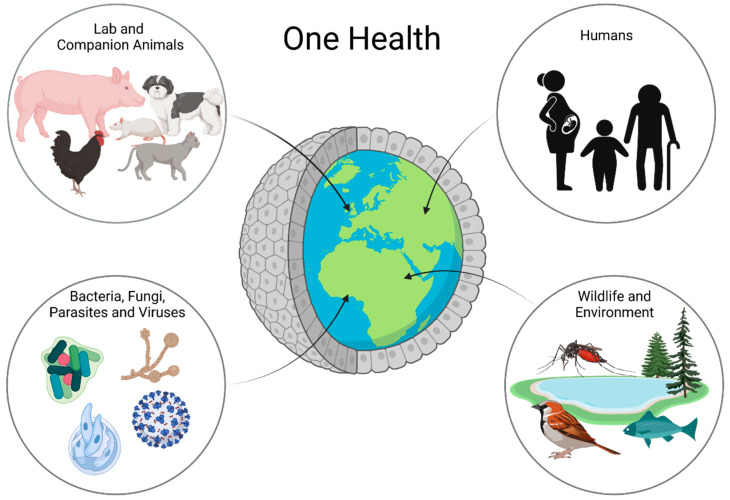
Organoids in One Health Research: Organoids are a possible way to work with all parts of One Health in one platform. Using organoids, one can learn about animal and human health and disease as well as interactions with the environment and bacteria, fungi, parasites, and even viruses.

In recent years, intestinal organoids have become increasingly important in research. They do not only represent a more complex system than classical two-dimensional cell cultures, but their three-dimensional nature also allows for the long-term maintenance and differentiation of many different cell types within one dish. Despite their complexity, intestinal organoids bear the advantage of only consisting of one layer of epithelial cells, thus putting the intestinal epithelial lining at the heart of the research. Intestinal organoids are not only valuable models for the investigation of complex diseases, such as IBD [64,65], but also represent a system which makes it possible to propagate pathogens in vitro, which previously could not be cultured, such as *Cryptosporidium* [66]. Beyond that, organoids even open up opportunities for precision medicine, as any effects can be studied in a patient-specific manner. Organoids can be the missing piece in the puzzle of performing research in a One Health context (Figure 2).

## 3. Organoids Modelling the Intestinal Epithelium

The mammalian intestines consist of the small intestine, i.e., duodenum, jejunum and ileum, and the large intestine, i.e., caecum and colon. There are fundamental differences between the small and large intestines, ranging from distinctive cell types over different tissue architectures to different physiological functions as a whole [67,68]. While all sections of the intestine contain certain cell types, such as stem cells, enteroendocrine cells, and goblet cells, and other cell types are only present in specific parts. M-cells, for instance, are only present in the epithelium on top of immune follicles, the intraintestinal lymphatic tissue, also known as gut-associated lymphatic tissue (GALT). There they interact with microbial antigens on their apical cell surface and then present these antigens on the basolateral cell surface to immune cells, thereby initiating an immunologic response [69]. An even more prominent example is Paneth cells in intestinal crypts, where they are intermingled with stem cells and pose an indispensable part of the so-called stem cell niche. These Paneth cells can only be found in crypts of the small intestine but not the colonic epithelium [70]. In 2019, van Es et al. reported that the depletion of Paneth cells from mouse intestines is leading to the adaptation and migration of enteroendocrine cells as well as tuft cells into the crypts in order to supply the stem cell niche with essential growth factors. This may be an alternative also for species in which the existence of Paneth cells has not yet been documented, as is the case for dogs and cats [71,72].

When culturing adult-stem-cell-derived organoids, many of the aforementioned characteristics can be recapitulated in vitro, starting from a single stem cell [30]. Usually, intestinal organoids represent a polarised epithelium of several different cell types, with the basolateral cell surface presented to the outside and the microvilli-bearing apical cell surface oriented towards the lumen side [73]. In 2021, a report highlighted the importance of using organoids from different organisms when it comes to drug toxicity and not simply extrapolating existing results to other species. Anti-cancer drugs have been tested in pig, monkey, and human intestinal organoids and demonstrated differing sensitivities between all three species [74]. Interestingly, Rosselot et al. showed that intestinal organoids even follow a circadian rhythm and that mouse and human organoids react differently to *C. difficile* toxin B depending on their circadian phase, which introduces a whole new level of complexity [75].

Standard intestinal organoids can also be used to model inflammatory bowel diseases. One study shows that human Crohn’s Disease (CD) patients have increased interleukin-28A (IL-28A) plasma levels, and organoids were used to model this system and its effects. When they applied IL-28A to human intestinal organoids, their barrier integrity was disrupted in a JAK-STAT-pathway-dependent manner, possibly modelling an important process in CD pathogenesis, as an impaired intestinal barrier is one major aspect of CD. In veterinary science, organoids recently helped to overcome the problem of not being able to propagate serotype I feline coronaviruses (FCoVs). Making this possible now allows for an in-depth functional analysis of the pathogenesis of feline infectious peritonitis and possible treatments [39].

However, to study gastrointestinal diseases using intestinal organoids, many applications depend on the ability to gain access to the apical cell surface on the inside of the organoids, which poses a major hurdle in disease modelling. In order to make the apical cell surface more accessible, several methods have been developed over the last few years:

### 3.1. Microinjection

Microinjection is a rather laborious method to gain access to the apical cell surface. It may require a lot of training of the experimenter and is not feasible for large-scale screening approaches. However, it is a well suitable method for studying host–microbe interactions. Hill et al. have established a microinjection approach using human intestinal organoids to study the host–microbe interactions of non-pathogenic *Escherichia coli* (*E. coli*). Microinjected *E. coli* were able to colonise the intestinal epithelium and establish a stable interaction between microbes and the host cells. This interaction was characterised by pronounced changes in the transcriptomic profile, epithelial proliferation, improved barrier integrity, and many more physiologically relevant adaptations [76]. This study was fundamental to a recent follow-up study by Abuaita and colleagues. They used different Salmonella serovars to find out whether known in vivo immune reactions could be modelled in vitro using intestinal organoids. As expected, different serovars led to different levels of immune responses, with the *Salmonella enterica* serovar Typhi infection leading to the weakest response, which is in accordance with its need to induce a weak host response in order to systemically infect the host. Additionally, many transcriptomic alterations induced by the three tested serovars were noticeable, which again highlights the usefulness of organoids for exploring new signalling pathways targetable in disease treatment and prevention [77]. This method cannot only be used to study bacteria–host interactions but is also applicable for the investigation of small parasitic organisms with the host epithelium, as shown by a model using *Cryptosporidium parvum* microinjection for infection and subsequent oocyst harvest [78]. However, as shown elegantly by the microinjection of *Lactobacilli*, when using pluripotent stem cell-derived intestinal organoids, one has to be cautious since the maturation stage of organoids can be increased using different culture media and can drastically influence the success of *Lactobacillus* colonisation of the organoid epithelium [79].

### 3.2. Apical-Out Organoids

Another useful method to gain access to the apical surface of the epithelium whilst not disrupting the three-dimensional structure of the organoids is the generation of so-called “apical-out organoids”. This method was first described in 2019 in a human enteroid model that appealingly demonstrated the importance of turning organoids inside out, providing the example of two different infection models. The rather simple method relies solely on the fact that organoids reverse their polarity once they are cultured floating in the culture medium without being embedded in an extracellular matrix [73]. A slightly modified version of this method was recently provided as a step-by-step protocol [80]. While *Salmonella* were used again to show their potential to infect the apical cell surface, organoids needed to be in their standard basal-out configuration to be infected by *Listeria monocytogenes* [73]. This study also used insights from research from 1994, which already used a three-dimensional model of canine cells (Madin–Darby canine kidney cells), which indicated an inherent function for beta 1 integrin in cell polarity [81]. Co et al. then demonstrated the importance of beta 1 integrin also for enteroid polarity, as applying a beta 1 integrin blocking antibody showed the same effects as the removal of extracellular matrix and led to organoid polarity reversal [73]. This is just one of many examples where first indications from animal cells give rise to novel approaches in more frequently used model systems, clearly highlighting the importance of interdisciplinary research.

Interestingly, intestinal apical-out organoids have been explored intensely in different animal species but not so much in mouse and human organoids over the last few years. A study using pig organoids analysed their potential to form apical-out organoids and set up functional readouts as fatty acid uptake and barrier integrity analyses [82]. There are several groups working on apical-out organoids for disease modelling in different contexts. For instance, porcine apical-out organoids were employed as an in vitro system to analyse the possibility of infecting organoids with the swine-enteric transmissible gastroenteritis virus (TGEV) and their immune response elicited by this virus [83]. Apart from using sheep gastrointestinal basal-out organoids for investigating the host–parasite interaction of *Teladorsagia circumcincta* with the epithelium, ovine apical-out organoids have also been tested in co-culture with *Salmonella enterica* serovar Typhimurium [84]. Meanwhile, chicken apical-out organoids have also proven to be a valuable tool for analysing different host–pathogen interactions. The protozoan *Eimeria tenella* can infect avian apical-out organoids just as well as the influenza A virus. This study also used *Salmonella enterica* as a bacterial example for avian gastrointestinal infection [85]. This is probably due to *Salmonella* being a facultative anaerobic bacterium relevant for the intestinal epithelium, as using obligate anaerobes such as *Fusobacterium* or *Clostridia* with low oxygen tolerance would not be compatible with the cultivation of apical-out organoids. However, especially these bacterial genera might be of interest for research in the future as they are frequently implicated in gastrointestinal diseases such as colon cancer and ulcerated regions in the human intestine [86] as well as in acute haemorrhagic diarrhoea syndrome (AHDS) in dogs [87]. Specifically, *Clostridia* might bear the risk of being a zoonotic bacterium in the context of One Health, as already outlined above. However, some *Clostridia*, such as *Clostridium hiranonis*, may also exert positive effects on gastrointestinal health. It was reduced in the dysbiosis index of dogs with chronic enteropathy in general [88] and, more specifically, in dogs with IBD [89]. *Clostridium hiranonis* possesses the ability to metabolise bile acids, and the dysregulation of bile acids has been associated with human IBD [90] and in dog enteropathies [91,92], which could potentially be modelled in vitro in the future.

### 3.3. Organoid-Derived Monolayers

Since handling organoids can be tedious and many standard assays are not adapted to three-dimensional structures, great efforts have been made during the last few years to find a way to reduce the complexity of the organoid system while simultaneously maintaining as many advantages of the organoids as possible. One way to do so is the use of organoid-derived monolayers (ODMs), which serve as a model of an intact intestinal barrier [93]. Classical two-dimensional in vitro models such as the Caco-2 cell system are most frequently used for drug screening and basic research. However, Caco-2 cells are derived from cancer cells and lack some possibly important epithelial enzymes and transporters [94]. Organoid-derived monolayers can be analysed, such as standard two-dimensional cell cultures, and have the advantage that they consist of several different cell types. Additionally, you can prepare them from whatever species you are able to culture organoids from. ODMs can thus be of great help in exploring transepithelial transport of nutrients, damage to the epithelial barrier integrity or similar approaches.

Human intestinal organoid-derived monolayers have been previously used as a model for pharmacokinetics and toxicology. In two-dimensional monolayers, the drug-metabolising enzyme CYP3A4 and several transporters were upregulated compared to Caco-2 cells and intestinal epithelial cells derived from induced pluripotent stem cells and resembled the adult duodenum more closely. These papers also showed the existence of all major differentiated cell types (enterocytes, enteroendocrine cells, goblet cells, and Paneth cells) in these monolayers, while stem cells decreased over time [95,96]. Another study demonstrated the ability of differentiated monolayers to actively transport ions (sodium, potassium, and chloride) and that the hormones serotonin and GLP-1 are produced by epithelial cells [97]. The functional transport of chloride ions has also been shown in porcine organoid-derived monolayers consisting of enterocytes, goblet cells and enteroendocrine cells [98]. Likewise, canine organoids have been used to create transwell-based ODMs that build up a functional barrier that can be used for dog gut research [99]. Aside from functional characteristics, ODMs have also been developed much further as co-culture models with bacteria. Mayorgas et al. used human ODMs as a proxy for the infection with invasive *E. coli* [100]. A slightly more complex system has been introduced by Sasaki et al. [101]. Here, ODMs are produced on transwell inserts. Once these monolayers reach confluence, the transwell chamber is sealed by a butyl rubber plug. This leads to an anaerobic apical chamber, while the bottom chamber, which is in contact with the basolateral cell surface, still has continuous access to oxygen. To test the so-called Intestinal Hemi-Anaerobic Coculture System (iHACS), the apical chambers were challenged with four different anaerobic bacterial strains (*Bifidobacterium adolescentis*, *Bacteroides fragilis*, *Clostridium butyricum*, and *Akkermansia muciniphila*) and showed the possibility for bacterial survival and propagation over five days of co-culture. This complex example showcases the possibility of using monolayers for bacterial co-culture and possible invasion analyses or co-cultures with commensal bacteria.

## 4. Limitations

Despite offering outstanding new possibilities for research, e.g., in vitro analysis of physiologic processes, disease modelling and genetic manipulation, organoids also confront researchers with some difficulties and limitations. For example, imaging approaches are more difficult to carry out compared to classical 2D cell culture approaches due to the three-dimensional structure of organoids and the resulting thickness of the sample in whole-mount stainings. However, imaging technology is gradually becoming better, and as confocal laser scanning microscopy (CLSM) is available virtually everywhere, this problem is also becoming smaller. New imaging techniques, such as spinning disk confocal imaging, offer new possibilities, especially for live-cell imaging, as the imaging process itself becomes much faster than in classical CLSM [102]. To overcome the problem of imaging depth, several different tissue-clearing methods have been developed [103,104]. These protocols enable the optical clearing of whole organoids or in vitro 3D tissues for considerably improved clarity and easier imaging of whole-mount samples.

Another difficulty is the batch-to-batch variations of the conditioned media, media supplements, and inhibitors. As organoids require a complex mix of stimulatory and inhibitory components in the medium to simulate the stem cell niche and/or provide the right cues for cell differentiation, all these supplements need to be of high and standardised quality. Chemically synthesised molecules tend not to be a problem as they are of extremely high and pure quality and undergo the appropriate quality checks. However, many labs rely on self-produced conditioned media as supplements for organoid culture media. These conditioned media can substantially vary, depending on the production process, hence skewing the results and hindering reproducibility, even though a report shows that the conditioned media production appears to be reproducible from batch to batch across several different laboratories [105]. To overcome this problem, more cost-intensive, specially designed so-called “surrogate” proteins can be used at defined concentrations [106,107,108]. Another problem arising from organoid culture media is the variation of media composition between laboratories. While some laboratories still rely on the original culture media [29,30,36,47], certain media are available for driving organoid differentiation while simultaneously ensuring a certain level of stemness in the same dish in human and canine intestinal organoids [37,109] or promoting full differentiation, for example in liver organoids [41,110]. These differences require a highly transparent methodology to ensure reproducibility and highlight the need for standardisation, as outlined by Gabriel et al. [38].

Because organoids are a very complex 3D model, it can be hard to precisely identify the specific factors that provoke the observed changes. Organoids receive various cues from media components and also the extracellular matrix they are grown in that need to be integrated into a physiologic context within the organoid. Therefore, small deviations from standard parameters can provoke drastic changes in the organoids. Matrix proteins are a major part of this dilemma. Matrigel still is the most prominently used extracellular matrix for the cultivation of organoids. However, Matrigel and comparable alternatives are basement membrane extracts derived from Engelbreth–Holm–Swarm sarcomas from mice. Thus, using organoids for research is not necessarily reducing the need for animal experimentation, as large quantities of mice are needed to produce the required extracellular matrix. Additionally, since Matrigel is derived from animals, quality control is rather difficult, no standardised mixture of components is defined, and batch-to-batch variability can be problematic [111]. For the last few years, a lot of money has been invested to produce non-mammalian or even animal-free alternatives to Matrigel. These include but are not limited to peptide-based hydrogels [112], a highly tuneable polysaccharide-based synthetic hydrogel [113], plant-based nanofibrillar cellulose [114,115] and collagen derived from jellyfish [116]. Despite the availability of these mammalian-free matrices, many people have not adopted them in their labs because of time- and cost-intensive procedures.

## 5. Outlook

Organoids have one major advantage for future research, which is the opportunity to study diseases in a patient-specific manner. Organoids can be established from small biopsies of tissues and expanded in vitro for experimental needs. These can be used for patient-specific drug-screening approaches or the analysis of genetic risk factors for certain diseases [117,118]. However, increasing individuality inevitably leads to less standardised models. In future research, patient-specific models will always have to be analysed with reference to a specific benchmark, i.e., a standardised control sample. Companies such as HUB Organoids in the Netherlands are building large biobanks for human organoids where researchers can apply for licencing agreements in order to use specific healthy or diseased organoids for certain projects [119]. However, for animal research, no such biobank is available, most probably because the research community working with animal-derived organoids is only starting to develop and is still too small.

Organoids may also be used for therapeutic approaches in the future. Kruitwagen et al. showed hepatocyte transplantation in canines with the possibility of curing copper storage disease caused by a mutation of the copper metabolism-domain-containing 1 (COMMD1) gene. Liver organoids were established from COMMD1-deficient dogs, genetically modified to restore COMMD1 function and, subsequently, transplanted back into the dogs of origin. Despite the engraftment percentages being low, the transplanted cells were able to survive for more than two years after transplantation [41]. Sampaziotis et al. used cholangiocyte organoids for direct bile duct regeneration. Importantly, delivering organoids to regenerate damaged bile ducts was demonstrated in mice and humans. While live mice were injected with organoids, normothermic machine perfusion (NMP) was used for human studies, which allows for the physiological perfusion of organs ex vivo. This makes it much easier to control the environmental influences and analyse different parameters. Perfusing these livers with human cholangiocyte organoid cells led to successful engraftment in human bile ducts, demonstrating the proof of principle, that organoid transplantation is feasible in mice as well as humans in the future [120].

Another human/mouse study generated human islet-like organoids to pave the way for diabetes treatment via pancreas islet transplantation. Human induced pluripotent stem cells were differentiated to human islet-like organoids (HILOs) expressing insulin and subsequently transplanted into diabetic mice. These pancreatic island cells could re-establish glucose homeostasis and may be more effective than conventional glucose monitoring and insulin injections as island cells can take on multiple additional roles [121]. In 2020, Meran et al. used organoids from child patients with intestinal failure and expanded them in vitro. They subsequently seeded organoid cells on decellularised small and large intestinal matrices and transplanted these scaffolds into mouse kidney capsules or subcutaneous pockets. These grafts formed luminal structures after transplantation and demonstrated the possibility of re-populating decellularised scaffolds with in vitro expanded cells for transplantation [122]. Similarly, Sugimoto et al. grafted small intestinal organoids onto the surface of the colon. These grafts started to form villus structures and ameliorated the symptoms of small intestinal short bowel syndrome in rats by structurally replacing colon epithelium with small intestinal cells [123].

Scientists are making efforts worldwide to lift organoid technology to the next level, explore new model systems, and generate more meaningful and complex models that mimic in vivo physiology even closer. Recent improvements include organoids with increased complexity, as by Koike et al., who modelled endoderm organogenesis at the foregut–midgut boundary by differentiating human induced pluripotent stem cells. Using this model, they created organoids containing cells from the liver, bile ducts, pancreas, and duodenum organised in one single organoid [124].

Other approaches for combining several organs in one model system mostly go towards using organ-on-a-chip applications. Such a chip incorporates one or many microchannels to connect the chip with a capillary system. This allows for the injection of fluids in a controlled manner that also supports directed flow of a medium, as, for instance, the intestine also experiences in vivo. Chip technologies can also be upgraded with micro-sensors and pose an extremely complex system [125]. The advances in organoid technology, microfabrication, cell engineering, and imaging technologies have led organ-on-a-chip to become an innovative technology capable of reproducing physiological cell behaviours in vitro [126]. However, the use of species other than mice and humans for chip-based technologies is very limited, with only two reports. The combination of multiple interconnected organ-on-a-chip systems in a single platform is now bringing this technology to the next level that aims to emulate an entire biological entity that is seldom limited to a single organ termed “body-on-a-chip” [127,128]. Despite these new advancements, there is still a lot to learn from organoids themselves and together with organoids, organ-on-a-chip technologies will take science a step further to replace animal experimentation. The comparison of in vitro organ models from various species will also guide new ways to explore the interconnection of humans, animals, and the environment in the context of One Health and help to explore new treatment strategies for various diseases.

## 6. Conclusions

Organoids are a promising tool for modern research. The continuous developments of new technologies, co-cultures, and organoid manipulation techniques lead to constant advancement in the field and open up new possibilities for treatments. Organoids of the liver, pancreas, stomach, and intestine are currently the in vitro method of choice for gastrointestinal research. Learning from mouse and human studies, many organoid systems have been adapted to other species. People are just beginning to explore these organoids and their differences from well-characterised models. Animal organoids pose a valuable in vitro method to model and study diseases, test environmental irritants on different organ systems of various species and develop new therapeutics. Keeping this in mind, organoids are becoming increasingly important in regard to the 3Rs (and 4R concept) and One Health research.

## Data Availability

Not Applicable.
